# Supplementary material for: Discovery and application of insertion-deletion (INDEL) polymorphisms for QTL mapping of early life-history traits in Atlantic salmon
Source: BMC Genomics. 2010 Mar 8;11:156. doi: 10.1186/1471-2164-11-156 (PMC2838853; doi:10.1186/1471-2164-11-156)
Supplement: Additional file 2 — Information on developed 76 locus single-run INDEL panel in Atlantic salmon. Information on fluorescence labeling, primer concentrations, PCR pooling and links to alignments, INDEL motifs and GENESCAN (Burge and Karlin 1997) predictions of genes/exons are available in html format. [file 1471-2164-11-156-S2.ZIP › Additionalfile2/snpsummary18185.html]

```
Cluster 8990 Contig 1

prev  Summary    Contig List  next
```

Size of Consensus sequence = 755

Number of sequences = 6

Minimum redundancy = 2

Key

A gi|89835863|gb|DY695821.1|DY695821 EST\_ssal\_rgb2\_51560 ssalrgb2 mixed\_tissue Salmo salar cDNA Salmo salar cDNA clone ssal\_rgb2\_583\_177\_fwd 3', mRNA sequence  
B gi|24382807|gb|CA052564.1|CA052564 ssalrga507232 mixed\_tissue Salmo salar cDNA, mRNA sequence  
C gi|24389345|gb|CA059102.1|CA059102 ssalrga506352 mixed\_tissue Salmo salar cDNA, mRNA sequence  
D gi|85054541|gb|DW582719.1|DW582719 EST\_ssal\_rgb2\_47138 rgb2 Salmo salar cDNA clone ssal\_rgb2\_576\_060\_fwd 3', mRNA sequence  
E gi|89837502|gb|DY696685.1|DY696685 EST\_ssal\_rgb2\_52424 ssalrgb2 mixed\_tissue Salmo salar cDNA Salmo salar cDNA clone ssal\_rgb2\_584\_315\_fwd 3', mRNA sequence  
F gi|24350008|gb|CA044968.1|CA044968 ssalplnb505114 gut Salmo salar cDNA, mRNA sequence

3 SNPs detected

A B C D E F  cosegregation weighted

398 A - - - - A   3/3 100.00
399 A - - - - A   3/3 100.00
400 A - - - - A   3/3 100.00
